# The Aging Meninges: Anatomical and Immunological Remodeling as Emerging Diagnostic Biomarkers of Cerebral Senescence—A Narrative Review

**DOI:** 10.3390/diagnostics16030452

**Published:** 2026-02-01

**Authors:** Sabina-Ioana Chirica, Marius Valeriu Hînganu, Costin Chirica, Alexia Nădejde, Delia Hînganu

**Affiliations:** Department of Morpho-Functional Sciences I, Faculty of Medicine, Grigore T. Popa University of Medicine and Pharmacy Iasi, 700115 Iasi, Romaniamg-rom-34480@students.umfiasi.ro (A.N.); hinganu.delia@umfiasi.ro (D.H.)

**Keywords:** meninges, aging, cerebral senescence, diagnostic biomarkers, meningeal immunity, neuroimmunology, meningeal lymphatics, neurodegeneration, glymphatic system

## Abstract

**Background/Objectives**: Aging is a universal biological process characterized by progressive structural, cellular, and molecular alterations that contribute to functional decline and increased susceptibility to disease. In recent years, the cerebral meninges have emerged as an active regulatory interface between the central nervous system and peripheral tissues, with growing evidence supporting their involvement in immune surveillance, fluid homeostasis, and age-related neuropathology. **Methods**: This narrative review provides a comprehensive overview of the current state of knowledge regarding anatomical, immunological, and molecular remodeling of the aging meninges, with particular emphasis on features with potential diagnostic relevance for cerebral senescence. A systematic analysis of the literature was performed to summarize established and emerging meningeal biomarkers, including inflammatory mediators, immune cell population dynamics, vascular and lymphatic alterations, and extracellular matrix remodeling. **Results**: The available evidence indicates that age-related changes in meningeal immunity, lymphatic drainage, and fibrotic architecture are closely associated with impaired waste clearance, chronic low-grade neuroinflammation, and increased vulnerability to neurodegenerative processes. Importantly, this review highlights existing knowledge gaps and unresolved questions in the field, underscoring the need for integrated anatomical and molecular studies of meningeal aging. **Conclusions**: As a summary, by delineating current evidence and limitations, this work aims to provide a conceptual framework to guide future experimental and translational research focused on identifying novel diagnostic biomarkers of cerebral senescence.

## 1. Introduction

Aging is a complex and inevitable biological process characterized by progressive structural, cellular, and molecular alterations that affect all organ systems and ultimately contribute to functional decline and increased susceptibility to disease [1,2,3]. Over recent decades, substantial research efforts have focused on elucidating the mechanisms of biological aging and cellular senescence, leading to the identification of conserved molecular hallmarks such as chronic low-grade inflammation, immune dysregulation, extracellular matrix (ECM) remodeling, and impaired tissue repair [4,5,6,7]. These processes operate in a highly interconnected manner and collectively shape age-related vulnerability across tissues.

Within this broader biological framework, the central nervous system (CNS) undergoes profound age-related changes that include progressive neuroinflammation, altered immune surveillance, impaired clearance of metabolic waste, and increased susceptibility to neurodegenerative disorders [8,9,10]. For example, while the meningeal compartment typically serves as an immunological reservoir that facilitates the infiltration of immune cells into the brain parenchyma via lymphatic vessels during infection or inflammation, meningeal aging progressively impairs this mechanism. Consequently, this functional decline compromises the CNS ability to mount a coordinated immune response to pathological insults [11].

For many years, the CNS was considered an immunologically privileged site, largely shielded from systemic immune interactions by specialized anatomical barriers [12,13,14]. However, this traditional view has been fundamentally revised. Accumulating evidence now demonstrates that immune cells, cytokines, and vascular signaling pathways actively participate in CNS homeostasis, adaptation, and disease progression throughout the lifespan [15,16].

In this revised paradigm, the cerebral meninges have emerged as a critical anatomical and functional interface between the CNS and peripheral tissues. Far from being passive protective membranes, the meninges play essential roles in immune surveillance, cerebrospinal fluid (CSF) dynamics, neurovascular communication, and molecular exchange between the brain and the systemic circulation [17,18,19,20]. Their strategic location and complex cellular composition position them as a key regulatory compartment in both physiological and pathological contexts.

Anatomically, the meninges consist of three distinct layers—dura mater, arachnoid mater, and pia mater—each characterized by unique structural, vascular, and immunological properties [21,22,23]. The dura mater is a collagen-rich, highly vascularized tissue that hosts diverse immune cell populations and specialized vascular and lymphatic networks. The arachnoid mater forms a selective barrier that regulates molecular trafficking between the CSF and the dural compartment, while the pia mater closely follows the contours of the brain and participates in neurovascular and immune interactions at the cortical surface [24,25,26,27]. This layered organization underpins a high degree of functional specialization within the meningeal compartment.

Recent murine studies have shown the importance of meningeal lymphatic vessels in maintaining CNS homeostasis. These vessels contribute to CSF drainage, immune cell trafficking, and clearance of metabolic waste products from the brain [28,29,30,31]. Together with the glymphatic system, which facilitates fluid exchange between CSF and interstitial fluid (ISF) along perivascular pathways, meningeal lymphatics form an integrated clearance network essential for normal brain function [32,33,34,35]. Disruption of this glymphatic–meningeal lymphatic axis has been increasingly linked to cognitive decline and the accumulation of neurotoxic proteins during aging.

With advancing age, the meninges undergo significant anatomical, immunological, and molecular remodeling. Documented age-related changes include shifts in immune cell composition, increased expression of pro-inflammatory mediators, impaired lymphatic drainage, vascular dysfunction, and progressive remodeling of the ECM, particularly within the dura mater [36,37,38,39,40]. Fibrotic changes and altered collagen organization may reduce tissue compliance and permeability, thereby compromising fluid dynamics and immune cell trafficking. Importantly, these alterations do not occur uniformly across meningeal layers, suggesting region-specific vulnerability and distinct regulatory mechanisms [41,42,43].

In a healthy aging brain, neuroinflammaging is characterized by a persistent, low-grade, and sterile inflammatory state. This process is primarily mediated by the systemic generation of reactive oxygen species (ROS), as well as the chronic release of proinflammatory chemokines, cytokines, and secondary messengers [44]. Thus, these age-associated meningeal changes are increasingly recognized as contributors to persistent low-grade neuroinflammation, driven by chronic interferon gamma (IFN-γ)– and TNF-mediated signaling, impaired lymphatic clearance, and sustained activation of meningeal immune cells. Moreover, emerging data indicate that meningeal aging exhibits specific cellular and molecular signatures that distinguish it from generalized systemic senescence. Such features raise the possibility that the meninges may serve not only as passive indicators of CNS aging but also as active modulators of age-related brain dysfunction.

Despite rapid progress in the field, substantial gaps remain in our understanding of meningeal aging. While numerous biomarkers of aging have been described in peripheral tissues and within the brain parenchyma, their relevance, specificity, and diagnostic value at the meningeal level remain incompletely characterized. In particular, the relationships between meningeal immune remodeling, vascular and lymphatic dysfunction, ECM alterations, and their combined impact on CNS aging require further integrated investigation.

The aim of this narrative review is to provide a comprehensive overview of the current state of knowledge regarding anatomical, immunological, and molecular remodeling of the aging meninges, with particular emphasis on features that may serve as diagnostic biomarkers of cerebral senescence. By synthesizing existing evidence and identifying unresolved questions, this review was conceived as a foundational analysis to support ongoing and future anatomical and molecular investigations into meningeal aging, including doctoral-level research initiatives aimed at defining novel diagnostic and translational targets.

Where possible, this review prioritizes mechanistic insights derived from primary experimental and translational studies, including single-cell profiling, functional imaging, and interventional animal models, rather than relying solely on descriptive syntheses.

An overview of these interconnected processes and their diagnostic relevance is provided in the graphical abstract.

## 2. The Meninges: Anatomy, Functional Importance, and Knowledge Gaps

The cerebral meninges represent a complex, multilayered system that plays a fundamental role in maintaining CNS homeostasis. Traditionally regarded as passive protective membranes, the meninges are now recognized as dynamic structures involved in immune surveillance, CSF circulation, vascular regulation, and molecular exchange between the CNS and peripheral tissues. Their strategic anatomical position and cellular diversity place them at the intersection of neurobiology, immunology, and vascular biology, making them increasingly relevant in the context of brain aging and disease.

### 2.1. The Meninges as a Key Interface in Brain Health

The cerebral meninges consist of the dura mater and the leptomeninges, the latter comprising the arachnoid mater and pia mater. Together, these three layers form a continuous and highly specialized interface that surrounds the brain and spinal cord, providing both mechanical protection and functional regulation of CNS homeostasis. Far from acting as passive coverings, the meninges integrate structural, vascular, immunological, and molecular functions that are essential for normal brain physiology.

The dura mater is a dense, collagen-rich membrane composed of an outer endosteal layer adherent to the cranial bones and an inner meningeal layer closely associated with the arachnoid mater [21]. Its fibrous architecture confers mechanical strength and resistance to deformation, allowing it to protect the brain from external forces. Within the dural compartment lie the venous sinuses, which serve as major conduits for cerebral venous blood drainage and play a central role in CSF reabsorption through arachnoid granulations. Through these mechanisms, the dura mater contributes to the regulation of intracranial pressure and CSF homeostasis, processes that are critical for maintaining stable neural function [22]. In addition, the dura mater hosts an extensive vascular and lymphatic network and represents the principal site of immune cell residence within the meningeal system, positioning it as a key neuroimmune interface.

The arachnoid mater is a thin, primarily avascular membrane that separates the dura mater from the subarachnoid space (SAS). It is characterized by a complex network of trabeculae that span the SAS, anchoring the arachnoid to the pia mater and contributing to mechanical stabilization of the brain within the cranial cavity. The SAS contains CSF and large cerebral blood vessels, providing both cushioning and metabolic support to the underlying neural tissues [23,24]. Structurally, the arachnoid mater forms a selective barrier through tight and tricellular junctions, which restrict uncontrolled molecular and cellular trafficking between the CSF and the dural compartment. This barrier function is essential for maintaining the distinct biochemical environments of the CSF and meningeal tissues.

The pia mater constitutes the innermost meningeal layer and consists of a delicate sheet of flattened fibroblast-like cells that closely follows the contours of the brain surface, extending into sulci and fissures. Unlike the arachnoid mater, the pia mater is highly vascularized and intimately associated with cortical blood vessels. It contributes to neurovascular regulation, nutrient exchange, and immune surveillance at the cortical interface, thereby linking meningeal function directly to parenchymal physiology [25]. The pia mater also participates in regulating perivascular spaces, which are critical conduits for fluid exchange and are central to glymphatic function.

Importantly, the three meningeal layers do not function in isolation but form a structurally and functionally integrated system. Their coordinated interactions enable the meninges to support CNS protection while permitting regulated communication with the peripheral environment. This integration allows the meninges to serve as a dynamic boundary that adapts to physiological demands, such as changes in intracranial pressure, immune challenges, and metabolic load.

Recent advances in neurobiology have increasingly highlighted the meninges as an active regulatory interface rather than a passive barrier. They are now recognized as key participants in immune surveillance, CSF circulation, waste clearance, and neurovascular signaling. The strategic location of the meninges at the intersection of the CNS, vasculature, and immune system positions them as critical modulators of brain health across the lifespan.

From the perspective of aging and disease, the meningeal interface is particularly vulnerable to cumulative structural and functional changes. Alterations in meningeal integrity, vascular organization, and immune composition can disrupt CNS homeostasis and amplify age-related pathological processes. As such, the meninges represent a crucial anatomical substrate through which systemic factors, including immune aging and vascular dysfunction, can influence brain function.

In summary, the meninges constitute a multifunctional interface that integrates mechanical protection with vascular, immune, and metabolic regulation. Their layered organization and specialized properties enable precise control of CNS–periphery interactions. Understanding the meningeal system as a key interface in brain health provides a foundational framework for subsequent discussions on immune regulation, clearance mechanisms, fibrosis, and vascular aging, all of which are central to the concept of cerebral senescence.

### 2.2. Re-Evaluating the “Immune Privilege” of the Brain

The concept of immune privilege was initially introduced to describe anatomical sites capable of tolerating foreign antigens without eliciting classical immune responses, a phenomenon first observed in tissues such as the eye, testis, and brain [26,27,28]. For decades, the CNS was viewed through this conceptual framework, largely due to the presence of specialized anatomical barriers—most notably the blood–brain barrier (BBB)—and the apparent absence of conventional lymphatic drainage pathways. This interpretation fostered the long-standing belief that the CNS was largely isolated from systemic immune surveillance.

However, this paradigm has been progressively revised as advances in neuroimmunology, imaging, and molecular profiling have revealed active and highly regulated immune surveillance mechanisms operating at CNS interfaces. Rather than existing in a state of immune exclusion, the CNS is now recognized as maintaining a distinct immunological environment characterized by selective immune access, tightly controlled signaling, and context-dependent immune responses. In this updated framework, immune privilege is better understood as immune specialization rather than immune silence.

Seminal studies demonstrated that immune cells—including macrophages, dendritic cells (DCs), and lymphocytes—reside within the meningeal compartments and actively participate in both homeostatic and pathological processes [29,30,31]. These immune populations are not transient infiltrates but stable, functionally specialized residents that contribute to antigen sampling, cytokine signaling, and regulation of neuroimmune communication. Collectively, these findings established that immune privilege does not imply immune isolation but instead reflects a spatially and functionally regulated immune ecosystem adapted to the vulnerability and metabolic demands of neural tissue.

The meninges have consequently emerged as a central hub of CNS immune regulation. Positioned at the boundary between the brain parenchyma and the peripheral circulation, they serve as a primary site where immune cells interact with CNS-derived antigens, CSF, and vascular and lymphatic structures. The meninges support immune surveillance, defined here as the coordinated activity of border-associated macrophages, T lymphocytes, and antigen-presenting cells that sample CSF- and blood-derived antigens and regulate immune trafficking at the CNS borders.

Immune cell populations within the meninges display pronounced layer-specific distribution. The dura mater exhibits the greatest immune cell diversity, containing macrophages, B and T lymphocytes, DCs, mast cells, natural killer cells, and plasma cells [36,37,38]. These cells are strategically positioned near meningeal blood vessels, venous sinuses, and lymphatic channels, enabling efficient sampling of circulating and CSF-derived antigens as well as communication with peripheral lymphoid organs. Recent evidence has further highlighted the presence of organized immune niches within the dura mater, supporting local antigen presentation and humoral immune responses.

In contrast, the leptomeninges contain fewer immune cells, reflecting their distinct structural organization and limited direct vascular access. The arachnoid mater forms a selective barrier through tight and tricellular junctions that restrict uncontrolled molecular and cellular trafficking between the CSF and the dural compartment. The pia mater, while highly vascularized, maintains specialized interactions with the cortical surface and supports immune surveillance in close proximity to neural tissue without promoting widespread immune cell accumulation. The selective permeability of these layers ensures that immune responses at the CNS borders remain tightly regulated [39].

This compartmentalization underscores the functional specialization of meningeal layers in shaping CNS immune responses. By localizing immune activity primarily to the dura mater and restricting access to the leptomeninges and parenchyma, the CNS minimizes the risk of immune-mediated damage while preserving effective surveillance. Importantly, this layered immune architecture is dynamic and responsive to physiological and pathological cues, including aging, infection, and neurodegeneration.

Re-evaluating immune privilege through the lens of meningeal immunology has important implications for understanding brain aging. Age-related changes in meningeal immune cell composition, activation state, and trafficking patterns can shift the balance from regulated surveillance toward chronic low-grade inflammation. Such alterations may compromise barrier integrity, impair lymphatic drainage, and facilitate the spread of inflammatory signals into the brain parenchyma. Consequently, the meninges represent a critical anatomical and immunological substrate through which systemic immune aging can influence CNS function.

In summary, the modern concept of immune privilege recognizes the CNS as an immunologically active organ protected by specialized regulatory mechanisms rather than by isolation. The meninges occupy a central position in this system, orchestrating immune surveillance, tolerance, and communication at the CNS borders. Understanding how these finely tuned immune interactions change with age is essential for elucidating the mechanisms of cerebral senescence and for identifying meningeal immune features with potential diagnostic relevance.

### 2.3. The Glymphatic–Meningeal Lymphatic Axis

A major paradigm shift in neurobiology followed the identification of functional lymphatic vessels within the dura mater, overturning the long-standing view that the CNS lacks conventional lymphatic drainage. The discovery of meningeal lymphatic vessels expressing canonical lymphatic endothelial markers, including LYVE-1, PROX1, and podoplanin, provided direct anatomical and molecular evidence of a structured drainage system linking the CNS to peripheral lymph nodes [40]. These vessels are primarily located along dural sinuses and major meningeal arteries, forming an organized network that enables CSF outflow, immune cell trafficking, and antigen drainage toward deep cervical lymph nodes.

Subsequent studies expanded these observations by identifying specialized lymphoid and immune-associated structures within the dura mater. These findings revealed that the meningeal lymphatic system is closely integrated with local immune niches, supporting antigen presentation, immune surveillance, and communication between the CNS and the peripheral immune system [41]. Together, these discoveries redefined the meninges as an active immunological and clearance interface rather than a passive protective barrier.

In parallel with advances in meningeal lymphatic biology, the glymphatic system was described as a glial-dependent pathway facilitating the exchange of CSF and ISF along perivascular spaces within the brain parenchyma. This system relies on astrocytic endfeet expressing aquaporin-4 water channels, which promote convective fluid flux along arterial and venous pathways [42,43]. Through this mechanism, metabolic waste products—including amyloid-β, tau, and other neurotoxic solutes—are cleared from the brain interstitium and transported toward CSF compartments.

The functional integration of the glymphatic system with meningeal lymphatic vessels constitutes a unified clearance axis essential for CNS metabolic homeostasis. Glymphatic influx delivers CSF into the brain parenchyma, while meningeal lymphatics provide an efflux route for cleared solutes and immune cells. This coordinated system ensures efficient waste removal, regulation of interstitial composition, and immune surveillance under physiological conditions. Disruption at any point along this axis can therefore have widespread consequences for brain function.

Growing evidence indicates that aging profoundly impairs the efficiency of the glymphatic–meningeal lymphatic axis. Experimental and clinical studies have demonstrated that advancing age is associated with reduced CSF influx, diminished perivascular transport, and decreased lymphatic drainage capacity. These changes result in impaired clearance of metabolic waste and increased retention of neurotoxic proteins, processes strongly implicated in cognitive decline and neurodegenerative disease [32,33,34]. Importantly, age-related clearance deficits may precede overt neurodegeneration, suggesting that dysfunction of this axis represents an early event in cerebral aging.

Several mechanisms have been proposed to underlie age-related impairment of the glymphatic–meningeal lymphatic system. At the parenchymal level, altered astrocyte polarization and mislocalization of aquaporin-4 channels reduce glymphatic efficiency. At the meningeal level, lymphatic vessels exhibit reduced diameter, decreased contractility, and altered endothelial gene expression with age, all of which compromise drainage capacity. In addition, aging is associated with reduced responsiveness of lymphatic vessels to mechanical and inflammatory stimuli, further limiting adaptive clearance responses.

Structural and molecular alterations of the meninges play a critical modulatory role in this context. Age-related vascular dysfunction, immune remodeling, and ECM changes within the dura mater can directly influence lymphatic vessel patency and function. Fibrotic thickening and collagen accumulation may mechanically constrain lymphatic channels, while chronic low-grade inflammation can impair lymphatic endothelial integrity and signaling. These factors collectively weaken the coupling between glymphatic inflow and meningeal lymphatic outflow, particularly during aging.

The glymphatic–meningeal lymphatic axis is also closely linked to immune aging. Meningeal immune cells, including macrophages and T lymphocytes, regulate lymphatic vessel function through cytokine signaling. Age-related shifts toward pro-inflammatory immune profiles—characteristic of inflammaging—have been shown to impair lymphatic drainage and exacerbate clearance deficits. This establishes a feed-forward loop in which impaired clearance promotes inflammation, which in turn further disrupts lymphatic and glymphatic function.

From a translational and diagnostic perspective, dysfunction of the glymphatic–meningeal lymphatic axis represents a promising target for biomarker development. Advances in neuroimaging, including contrast-enhanced MRI and dynamic CSF tracer studies, have enabled indirect assessment of glymphatic and meningeal lymphatic function in humans [35]. Age-related alterations in tracer distribution, clearance kinetics, and meningeal signal intensity may provide measurable indicators of cerebral senescence and early neurodegenerative risk.

Collectively, these findings establish the glymphatic–meningeal lymphatic axis as a central regulator of CNS homeostasis whose integrity declines with age. Understanding how structural, vascular, immune, and ECM-related changes in the meninges influence this clearance system is essential for elucidating the mechanisms of cerebral aging. Moreover, integrating functional assessments of glymphatic and meningeal lymphatic activity with molecular and anatomical markers may support the development of novel diagnostic strategies aimed at detecting and monitoring age-related brain dysfunction.

### 2.4. Meningeal Fibrosis and Aging

The meninges are predominantly composed of collagen-rich connective tissue, making the organization and composition of their ECM critical determinants of structural integrity and functional performance. With advancing age, the meningeal layers—particularly the dura mater—undergo progressive ECM remodeling characterized by increased collagen deposition, altered fiber organization, enhanced cross-linking, and changes in elastin content. This process, commonly referred to as meningeal fibrosis, represents a hallmark of structural aging at the CNS borders and has important implications for tissue compliance, permeability, and cellular trafficking [45].

Comparative anatomical studies have demonstrated that the structural organization of the dura mater differs substantially between cranial and spinal regions, reflecting functional specialization and distinct mechanical demands. While the spinal dura mater exhibits a multilayered architecture with clearly defined cellular and fibrous compartments, the cranial dura mater consists of dense fibrous tissue interspersed with flattened fibroblast-like cells and abundant collagen fibers. Notably, collagen fibers within the cranial dura mater are arranged in concentric, interwoven, and multidirectional patterns rather than in parallel bundles, a configuration that confers mechanical resilience and resistance to deformation under physiological conditions. These baseline structural differences are important for interpreting age-related remodeling, as they influence how fibrotic processes manifest regionally.

Age-related structural changes in the dura mater have been documented in cadaveric, histological, and imaging-based studies. Several investigations have reported an increase in dural thickness associated with fibrotic degeneration and collagen accumulation, whereas others have identified regional thinning, particularly at the skull base and areas adjacent to venous sinuses. These seemingly divergent findings underscore the heterogeneity of meningeal remodeling and highlight the influence of anatomical location, local mechanical stress, and vascular proximity on age-related changes [46,47]. Rather than representing contradictory observations, such variability likely reflects spatially distinct remodeling trajectories within the meningeal compartment.

More recent histological and ultrastructural analyses have provided detailed insights into the microstructural evolution of the dura mater across the lifespan. In younger individuals, the collagenous matrix appears dense and well organized, with elastic fibers aligned in parallel with collagen bundles and a relatively uniform distribution of fibroblastic cells. In contrast, aging is associated with progressive disorganization of collagen fibers, reduced matrix coherence, fragmentation and loss of elastic fibers, and increased collagen cross-linking. These alterations result in a mechanically stiffer, less compliant meningeal tissue with altered viscoelastic properties. At the cellular level, age-related changes in fibroblast phenotype and activity, including a shift toward pro-fibrotic signaling, are thought to contribute to sustained ECM deposition and remodeling.

Beyond its structural consequences, meningeal fibrosis has important functional implications. Increased tissue stiffness and reduced permeability may impair several key processes, including CSF dynamics, immune cell trafficking, and lymphatic drainage. Fibrotic thickening of the dura mater may mechanically constrain meningeal blood and lymphatic vessels, thereby altering local hemodynamics and reducing the efficiency of fluid clearance pathways. Such effects are particularly relevant in the context of the glymphatic–meningeal lymphatic axis, where coordinated movement of CSF and ISF depends on compliant perivascular and meningeal environments.

Meningeal fibrosis may also influence the local immune microenvironment. The meningeal ECM provides a structural scaffold that shapes immune cell localization, migration, and retention. Fibrotic remodeling can disrupt stromal–immune cell interactions, promote immune cell sequestration, and amplify local inflammatory signaling. These changes may reinforce age-related immunosenescence and inflammaging within the meningeal compartment, creating a feed-forward loop in which inflammation and fibrosis mutually exacerbate one another [48].

Importantly, fibrotic remodeling of the meninges does not occur in isolation but is closely intertwined with vascular and lymphatic aging. Age-related endothelial dysfunction, altered shear stress, and chronic inflammatory exposure may promote fibroblast activation and ECM deposition, while fibrotic stiffening can, in turn, impair vascular and lymphatic function. This reciprocal relationship suggests that meningeal fibrosis represents a central integrative process linking structural, vascular, and immune aspects of cerebral aging.

Collectively, these observations indicate that meningeal fibrosis represents a prominent and underappreciated feature of brain aging. Understanding its extent, regional variability, and molecular drivers is essential for elucidating its contribution to cerebral senescence. Moreover, structural alterations of the meningeal ECM—such as changes in dural thickness, collagen organization, and tissue stiffness—may represent measurable features with potential diagnostic relevance. Advances in high-resolution imaging and biomechanical assessment raise the possibility that meningeal fibrosis could be incorporated into future diagnostic frameworks aimed at identifying and monitoring age-related neurological disorders.

### 2.5. Blood Circulation of the Cerebral Meninges: Vascular Organization, Neuroimmune Functions, and Aging-Relevant Diagnostic Readouts

The cerebral meninges are supplied by a highly specialized vascular network that differs substantially from the intraparenchymal cerebral microcirculation. Beyond nutrient delivery and venous drainage, meningeal blood vessels provide a structural scaffold for immune surveillance, regulate barrier properties at the CNS borders, and interact functionally with CSF outflow pathways and dural lymphatics. Because many of these processes undergo measurable remodeling with age, meningeal blood circulation has increasing relevance for biomarker discovery in cerebral senescence.

Arterial supply and layer-specific vascular architecture. The dura mater is the most richly vascularized meningeal layer. Its arterial supply arises predominantly from branches of the external carotid system, particularly the middle meningeal artery (MMA), with additional contributions from occipital and ascending pharyngeal branches; region-specific anastomoses link dural arteries to adjacent cranial and orbital circulations. Contemporary anatomical syntheses, driven in part by clinical interest in MMA embolization, have emphasized the complexity and heterogeneity of dural arterial territories and the proximity of major branches to cranial nerves and skull base structures, highlighting that “dural vasculature” is not a uniform entity but an anatomically compartmentalized system [49]. In contrast, the leptomeninges (arachnoid and pia) host a more delicate vascular organization: pial vessels accompany the cortical surface and bridge to penetrating vasculature, while the arachnoid itself is largely avascular and functions primarily as a barrier that separates the vascularized dura from the CSF-filled SAS [50,51].

Venous sinuses as more than passive drains. Meningeal venous circulation is dominated by the dural venous sinuses, which collect blood from bridging veins and ultimately drain to the internal jugular system. Importantly, the sinus walls are not inert conduits; they constitute an endothelialized stromal niche embedded in dura that supports antigen sampling and immune cell interactions. A key advance in the last five years has been the functional characterization of dural sinuses as a neuroimmune interface in which CSF-derived antigens accumulate near the sinus microenvironment and are captured by antigen-presenting cells, enabling T cell surveillance and local immune programming [52]. Complementing this view, later work has described discrete perivascular lymphoid structures within dura that support local humoral responses, reinforcing the concept that meningeal venous anatomy is tightly coupled to immune organization [53]. These findings are directly relevant to aging because immunosenescence and “inflammaging” can reshape the cellular composition and cytokine milieu of precisely these perivascular niches, with downstream consequences for clearance and barrier function.

Barrier features and exchange properties of dural vessels. Compared with the parenchymal BBB, meningeal vascular beds—especially within dura—exhibit distinct permeability features, and the dural compartment contains abundant fenestrated vasculature that favors exchange between blood and the meningeal stroma [51,53]. This difference is critical for understanding why inflammatory mediators and immune cells may be preferentially recruited or retained in the dura during aging, and why vascular-derived signatures in dura may diverge from BBB-centered biomarkers. Reviews focusing on BBB aging emphasize endothelial senescence, oxidative stress, and junctional alterations as drivers of increased permeability and neurovascular dysfunction [54]. While these syntheses are largely centered on brain microvessels, the same endothelial aging programs (reduced vasoprotective signaling, heightened inflammatory responsiveness, impaired glycocalyx function) provide a mechanistic framework for understanding age-related dysregulation of meningeal endothelium and sinus-associated stromal niches, particularly under systemic pro-inflammatory pressure.

Coupling to lymphatic and CSF clearance pathways—an aging-sensitive interaction. Meningeal blood circulation does not operate independently from CSF outflow and dural lymphatic function. Dural vessels and venous sinuses are spatially and functionally integrated with meningeal lymphatic networks and skull–meninges interfaces that coordinate immune trafficking and clearance [50,54]. Aging perturbs this integrated system through immune-driven signaling and ECM remodeling. For example, elevated meningeal interferon-gamma (IFN-γ) signaling in aged animals has been shown to impair meningeal lymphatic drainage, linking immune activation near meningeal vasculature to functional clearance deficits [55]. In parallel, recent evidence indicates that age-related dural ECM remodeling (including peri-lymphatic collagen accumulation) compromises lymphatic function and contributes to CSF clearance impairment, suggesting a mechanical and stromal component that can secondarily influence vascular compliance and local hemodynamics [56,57]. Taken together, these pathways support the idea that vascular aging in meninges is best interpreted as a composite phenotype involving endothelial–immune–stromal interactions rather than a purely hemodynamic change.

Diagnostic outlook: imaging-accessible readouts and candidate biomarker domains. From a Diagnostics perspective, meningeal blood circulation is attractive because it can be interrogated indirectly in vivo. High-resolution MRI sequences and contrast kinetics can capture signal changes within dural and perisinus compartments that reflect vascular permeability, stromal expansion, or altered fluid distribution. Notably, recent prospective imaging work has investigated the temporal distribution of contrast agent signal across dura mater, SAS, and perivascular regions—compartments implicated in glymphatic and meningeal lymphatic function—supporting the feasibility of dynamic imaging paradigms for meningeal assessment in humans [58,59,60]. Translationally, the most plausible biomarker directions include: (i) vascular integrity markers (endothelial activation molecules, circulating endothelial microparticles, senescence-associated secretory signatures); (ii) immune–vascular coupling markers (perisinus cytokine profiles, IFN-γ–linked panels, B cell/plasma cell activity in venous-plexus hubs); and (iii) structure–function imaging composites integrating dural thickness/ECM features with vascular/perfusion readouts. Importantly, these domains align with the broader premise of this review: meningeal aging may provide diagnostically meaningful signatures that precede or accompany parenchymal neurodegenerative change, particularly when measured as integrated vascular–immune–stromal phenotypes [61,62].

## 3. The Role of the Meninges in Age-Related Decline

Aging is accompanied by a progressive decline in immune system efficiency and the emergence of a chronic, low-grade inflammatory state commonly referred to as inflammaging. This systemic phenomenon is characterized by sustained elevation of pro-inflammatory mediators, dysregulated innate and adaptive immune responses, and impaired resolution of inflammation. Within the CNS, inflammaging contributes to persistent neuroinflammation, altered neuronal–glial communication, impaired tissue homeostasis, and increased vulnerability to neurodegenerative disorders. As an anatomical and functional interface between the CNS and the peripheral immune system, the meninges are particularly sensitive to these age-related immunological alterations and play a pivotal role in translating systemic immune aging into CNS dysfunction [63].

The meningeal compartment occupies a strategic position at the CNS borders, where immune, vascular, and CSF-associated signals converge. This location renders the meninges uniquely responsive to age-related changes in circulating immune cells, cytokines, and metabolic factors. Unlike the brain parenchyma, which is protected by the BBB, the meninges—especially the dura mater—are directly exposed to systemic immune influences. Consequently, age-related immune dysregulation is often first detected or amplified at the meningeal level, positioning the meninges as an early responder and potential driver of CNS aging.

Age-related changes in the meninges extend well beyond gross structural remodeling and encompass complex shifts in immune cell composition, activation state, and functional capacity. With advancing age, the balance between immune surveillance and immune tolerance within the meningeal compartment becomes progressively disrupted. Resident immune cells, including macrophages, T lymphocytes, and B cells, exhibit altered phenotypes characterized by increased pro-inflammatory signaling, reduced regulatory function, and diminished responsiveness to homeostatic cues. These changes collectively contribute to a meningeal microenvironment that favors chronic inflammation over tightly regulated immune monitoring.

Alterations in meningeal immunity have important consequences for immune surveillance and inflammatory signaling within the CNS. Under physiological conditions, meningeal immune cells participate in antigen sampling, cytokine-mediated communication, and coordination of immune responses without provoking widespread inflammation. During aging, however, sustained activation of these cells can lead to excessive production of inflammatory mediators, such as interferon-γ, tumor necrosis factor, and interleukins, which may diffuse into adjacent compartments or influence signaling across meningeal barriers. Such changes can indirectly affect neuronal activity, synaptic plasticity, and glial function, thereby linking meningeal immune aging to parenchymal dysfunction.

Communication between the meninges and peripheral lymphoid organs is also altered with age. The meninges serve as a key relay station for immune cell trafficking and antigen drainage via meningeal lymphatic vessels. Age-related impairment of lymphatic function, combined with immune cell dysregulation, can disrupt this communication axis, leading to reduced clearance of immune cells and inflammatory mediators from the CNS borders. The resulting accumulation of immune-derived signals within the meningeal compartment may further exacerbate local inflammation and impair CNS homeostasis.

Importantly, meningeal aging is a multifactorial process in which immune alterations interact with vascular dysfunction, ECM remodeling, and impaired fluid clearance. Chronic inflammation can promote fibrotic changes within the dura mater, alter vascular permeability, and compromise lymphatic drainage, while structural and vascular changes can, in turn, reinforce immune dysregulation. This bidirectional interplay suggests that the meninges function as an integrative platform where multiple hallmarks of aging converge and amplify one another.

The consequences of meningeal aging extend to the regulation of CSF dynamics and waste clearance. Inflammatory and structural changes within the meninges can impair the efficiency of the glymphatic–meningeal lymphatic axis, reducing the removal of metabolic byproducts and neurotoxic proteins from the CNS. Impaired clearance, coupled with persistent inflammatory signaling, creates a permissive environment for the accumulation of pathological proteins and the initiation or acceleration of neurodegenerative processes.

From a systems-level perspective, the meninges can thus be viewed as both sensors and effectors of age-related decline. They sense systemic immune aging through direct exposure to circulating factors and respond by remodeling their immune, vascular, and stromal components. These responses, while initially adaptive, may become maladaptive over time, contributing to a chronic state of meningeal dysfunction that undermines CNS resilience.

Collectively, these observations position the meninges as a critical mediator of age-related CNS decline. Rather than serving solely as passive boundaries, the meninges actively shape the trajectory of brain aging through their roles in immune regulation, vascular signaling, and fluid homeostasis. Understanding how age-related alterations in meningeal biology contribute to neuroinflammation, impaired clearance, and neurodegeneration is essential for elucidating the mechanisms of cerebral senescence. Moreover, because many of these changes occur at accessible CNS interfaces, the meninges represent a promising target for the identification of early diagnostic markers and potential intervention points in age-related neurological disorders. Understanding meningeal immunosenescence is therefore critical for elucidating the mechanisms linking aging to CNS dysfunction (Table 1).

To avoid ambiguity and to provide mechanistic clarity, the major meningeal immune cell populations discussed throughout the manuscript, along with their age-related functional changes and diagnostic relevance, are summarized in Table 1.

### 3.1. Immunosenescence of Meningeal Cells and Chronic Inflammation

Immunosenescence is characterized by a gradual deterioration of both innate and adaptive immune responses, accompanied by persistent low-grade inflammation. In the meninges, this process manifests as quantitative and qualitative changes in resident and recruited immune cell populations, as well as altered cytokine and chemokine signaling. Together, these changes contribute to a pro-inflammatory microenvironment that may disrupt CNS homeostasis.

Macrophages represent the most abundant immune cell population within the meningeal compartments. These cells, collectively referred to as border-associated macrophages (BAMs) or CNS-associated macrophages, reside at CNS interfaces including the meninges, perivascular spaces, and choroid plexus. Under homeostatic conditions, BAMs contribute to immune surveillance, phagocytosis of cellular debris, and maintenance of tissue integrity [79,80].

Aging is associated with marked alterations in meningeal macrophage populations. Studies employing single-cell and transcriptomic approaches have demonstrated an increased contribution of monocyte-derived macrophages with advancing age. These cells, often referred to as disease inflammatory macrophages, exhibit a pro-inflammatory phenotype characterized by elevated cytokine production and reduced homeostatic function. In contrast, embryonically derived macrophage populations may retain distinct, potentially neuroprotective roles, highlighting functional heterogeneity within the aging meningeal macrophage pool.

Adaptive immune cells also undergo significant age-related changes within the meninges. T lymphocytes, including γδ T cells and αβ T cells, migrate to the meningeal compartment via specialized vascular channels connecting the bone marrow to the dura mater. Under physiological conditions, meningeal T cells play important roles in regulating cognitive function, behavior, and immune surveillance. With aging, however, alterations in the stromal and vascular microenvironment promote the accumulation and retention of T cells within the meninges [81].

Experimental studies have demonstrated that aging is associated with an expansion of meningeal T cell populations and increased production of pro-inflammatory cytokines, particularly interferon-γ. Elevated cytokine levels within the dural compartment have been shown to impair meningeal lymphatic drainage and disrupt CSF outflow, thereby linking immune dysregulation to functional impairment of CNS clearance mechanisms [82].

B cells and plasma cells also contribute to age-related meningeal immune remodeling. The meninges represent a site of local antibody production and immune cell activation, particularly within dural lymphatic-associated niches. Aging-related changes in B cell composition and function may further amplify inflammatory signaling and influence CNS immune responses, although these mechanisms remain incompletely characterized.

### 3.2. Consequences of Meningeal Immunosenescence for CNS Homeostasis

The cumulative effects of meningeal immunosenescence extend beyond local inflammation and have systemic consequences for CNS function. Persistent inflammatory signaling within the meningeal compartment can influence neuronal activity, synaptic plasticity, and neurovascular regulation. Moreover, impaired meningeal lymphatic function resulting from immune-mediated remodeling may reduce the clearance of immune cells, cytokines, and metabolic waste from the CNS [83].

These processes collectively contribute to a feed-forward cycle in which inflammation, impaired clearance, and tissue dysfunction reinforce one another during aging. Importantly, emerging evidence suggests that meningeal immune alterations precede or accompany early stages of neurodegenerative disease, raising the possibility that meningeal immunosenescence represents an early and accessible indicator of cerebral aging [81,82,83,84].

From a diagnostic perspective, age-related changes in meningeal immune cell populations, cytokine profiles, and lymphatic function may serve as measurable biomarkers of cerebral senescence. Characterizing these alterations in greater detail may provide valuable insights into the timing, progression, and heterogeneity of brain aging, as well as inform the development of novel diagnostic and monitoring strategies for age-related neurological disorders.

### 3.3. A Dural Sinus–CSF Etiopathogenic Axis in Meningeal Senescence

Accumulating evidence supports the view that meningeal aging is not solely a diffuse process of “inflammaging” and fibrosis, but also reflects dysfunction of anatomically constrained neuroimmune and clearance hubs. Among these, the dural venous sinuses represent a particularly plausible epicenter for age-related meningeal dysfunction because they reside at the intersection of (i) venous outflow, (ii) CSF egress routes, (iii) dural stromal remodeling, (iv) meningeal lymphatics, and (v) immune surveillance niches. Conceptually, this supports a dural sinus–CSF etiopathogenic axis, in which age-related changes at the sinus-adjacent microenvironment initiate and amplify a feed-forward cycle of impaired clearance, chronic inflammation, and stromal remodeling that collectively define functional meningeal senescence.

#### 3.3.1. The Dural Sinus as a Neuroimmune Interface Exposed to CSF-Derived Signals

Rather than acting as inert venous conduits, dural sinuses constitute complex endothelial–stromal structures embedded within dura mater and closely associated with immune aggregates. Elegant mechanistic work has demonstrated that the dural sinuses operate as a neuroimmune interface, where immune cells—especially T cells—survey CNS- and CSF-derived antigens under steady-state conditions, supported by a specialized stromal niche comprising endothelial and mural components [85]. This positioning has important implications for aging: if antigen drainage and sampling occur preferentially at the sinus-adjacent dura, then immune remodeling and stromal changes in this compartment may have disproportionate effects on CNS border homeostasis.

A second line of evidence reinforcing this hub concept comes from the description of dural-associated lymphoid organization around venous plexi. Venous-plexus-associated lymphoid “hubs” in the dura have been shown to sample antigens and support local humoral responses, indicating that the sinus/plexus neighborhood is a privileged site for immune activation and memory formation [86]. Together, these findings motivate the idea that aging-related immune shifts (e.g., altered T cell retention, skewed cytokine production, increased B cell/plasma cell activity) can directly reshape the sinus-adjacent immune microenvironment and thereby influence meningeal function.

#### 3.3.2. CSF Egress Routes Converge at the Dura–Arachnoid–Sinus Interface

The axis also requires an anatomical logic for CSF–sinus coupling. Classical physiology recognized that CSF outflow occurs through arachnoid villi and granulations projecting into dural sinuses, enabling CSF passage from the SAS to venous circulation. Contemporary work has refined this picture by demonstrating that arachnoid granulations are not simple “valves,” but contain internal channel networks that communicate with perisinus spaces and the dura–arachnoid stroma, supporting the idea of structured trans-arachnoidal flow and neuroimmune roles [87]. This provides a modern substrate for a dural sinus–CSF axis: CSF-derived solutes and antigens can reach sinus-adjacent spaces where immune cells and stromal elements are positioned to sense, filter, and respond.

Importantly, the dural sinus region is also spatially aligned with meningeal lymphatics, which are often concentrated along dural sinuses and major meningeal vessels. Thus, the sinus neighborhood may function as a triangular junction connecting venous drainage, CSF transit, and lymphatic efflux. In practical terms, any age-related disruption in this neighborhood may simultaneously impair venous-associated immune surveillance, reduce CSF outflow efficiency, and weaken lymphatic drainage—three processes that converge on waste clearance and inflammatory tone.

#### 3.3.3. CSF Aging Transforms the Sinus-Adjacent Niche Through Immune Remodeling and Cytokine Signaling

A central mechanistic driver of this axis is age-related immune remodeling within the dura. In aged organisms, meningeal immune composition shifts toward increased T cell accumulation and a pro-inflammatory cytokine milieu. Critically, age-related impairment of meningeal lymphatic drainage has been causally linked to immune changes: single-cell profiling and functional experiments demonstrated that increased meningeal IFN-γ signaling (associated with T cell accumulation) drives lymphatic endothelial responses and impairs CSF drainage, while IFN-γ neutralization can alleviate age-related lymphatic dysfunction [88]. This places cytokine signaling at the heart of the etiopathogenic loop: immune aging → cytokine elevation → lymphatic/clearance impairment → retention of inflammatory mediators → further immune activation.

From an axis perspective, the dural sinus neighborhood is an ideal site for such a loop to develop because it hosts immune niches [84,85,86], receives CSF-derived antigens/solutes [18], and lies near lymphatic pathways that can be suppressed by pro-inflammatory signaling [87]. Therefore, aging can shift this region from regulated surveillance into a state of chronic immune activation and reduced drainage capacity—an operational definition of meningeal senescence.

#### 3.3.4. Stromal Fibrosis and ECM Remodeling: A Mechanical Amplifier of Clearance Failure

Immune-driven changes are unlikely to be the sole contributor. Aging also remodels dura mater through fibrosis and ECM alterations. Recent evidence identifies ECM remodeling in aged dura as a key driver of CSF clearance deficits: peri-lymphatic collagen accumulation disrupts lymphatic function, and mechanistic work implicates pro-fibrotic signaling pathways (including transforming growth factor-beta (TGF-β)-linked regulation in dural fibroblasts) as contributors to this remodeling [88]. These data strongly support a “mechanical amplifier” concept within the axis: once fibrosis and collagen cross-linking progress around sinus-adjacent lymphatic and perisinus spaces, compliance decreases and fluid transit becomes less efficient, thereby worsening stagnation and inflammatory retention.

This is particularly relevant to the sinus-adjacent dura, where the geometry of perisinus spaces, arachnoid granulations, and lymphatic channels may make the system sensitive to small changes in stiffness and microanatomy. Thus, even modest fibrotic remodeling may produce a disproportionate functional penalty at this anatomical bottleneck, magnifying age-related clearance impairment.

#### 3.3.5. A Feed-Forward Etiopathogenic Loop: From Impaired Drainage to Parenchymal Vulnerability

Integrating these findings, the dural sinus–CSF etiopathogenic axis can be summarized as a feed-forward loop:

- Aging remodels the dural sinus neighborhood (immune composition shifts; cytokine tone rises; stromal/ECM remodeling progresses) [88,89];
- CSF outflow and lymphatic drainage become less efficient, particularly in sinus-adjacent pathways and perisinus compartments [88,89,90];
- Retention of CSF-derived solutes, antigens, and inflammatory mediators increases, further stimulating local immune activation at the neuroimmune interface [85,86];
- Local inflammation and fibrosis reinforce each other, consolidating a senescent meningeal phenotype with impaired barrier/clearance performance [89].

Over time, parenchymal vulnerability rises indirectly, because impaired waste clearance and sustained border inflammation are linked to neuroinflammation and neurodegenerative risk (conceptually consistent with the broader glymphatic–meningeal lymphatic framework) [90,91].

A key strength of this axis is that it unifies vascular/venous anatomy, CSF transit, lymphatic outflow, immune niches, and fibrosis into one coherent pathogenic framework—precisely the kind of integrative model that can guide biomarker development and hypothesis-driven studies.

#### 3.3.6. Diagnostic and Translational Implications: Imaging-Accessible Targets and Biomarker Logic

From a diagnostic standpoint, the dural sinus–CSF axis represents a promising target because it may be probed in vivo using imaging paradigms that capture dural/perisinus enhancement patterns and clearance kinetics. Dynamic contrast-enhanced MRI has been used to assess meningeal lymphatic vessels—particularly in parasagittal dural spaces where lymphatics are prominent—supporting feasibility of quantifying meningeal drainage behavior [92]. In addition, dynamic intravenous contrast-enhanced MRI has been reported to visually evaluate impaired drainage of the glymphatic–meningeal lymphatic system in clinical populations (e.g., high-burden cerebral small vessel disease), suggesting translational relevance of contrast-based clearance phenotypes [91]. Although these approaches do not measure “sinus–CSF coupling” directly, they provide practical readouts of the downstream functional consequence central to the axis: impaired clearance within sinus-adjacent meningeal compartments.

Mechanistically informed biomarkers could include composite signatures combining: (i) perisinus structural markers (dural thickening, fibrosis proxies, parasagittal dural space metrics); (ii) functional drainage markers (contrast enhancement timing/clearance curves reflecting meningeal lymphatic and CSF transit); and (iii) immune activation signatures (cytokines linked to drainage impairment such as IFN-γ–associated panels) [88,91]. Finally, integrative conceptual reviews emphasize that glymphatic-driven mobilization of brain “waste” toward immunologically active borders encodes neuroimmune communication, supporting the plausibility of border-based biomarkers as early indicators of CNS aging [40]. In sum, framing meningeal senescence through a dural sinus–CSF axis yields testable predictions for imaging and molecular profiling and provides a structured scaffold for future translational studies.

### 3.4. Translational Considerations and Model Limitations

Much of the mechanistic insight into meningeal immune remodeling, lymphatic dysfunction, and stromal fibrosis during aging derives from experimental animal models, predominantly mice. While these models have been instrumental in defining causal relationships and cellular mechanisms, important anatomical and physiological differences between rodents and humans—particularly in meningeal thickness, immune organization, and lifespan-related dynamics—must be considered when extrapolating findings. Human data are currently more limited and rely largely on post-mortem analyses and advanced neuroimaging approaches, underscoring the need for carefully designed translational studies.

## 4. Future Perspectives and Diagnostic Outlook

The growing recognition of the cerebral meninges as an active regulatory interface between the CNS and peripheral tissues has opened new avenues for understanding brain aging and its associated disorders. Accumulating evidence reviewed in this article indicates that meningeal aging is characterized by coordinated anatomical, immunological, and molecular remodeling, affecting immune surveillance, fluid clearance, and ECM organization. Importantly, many of these changes exhibit distinct spatial and cellular patterns that differentiate meningeal aging from generalized systemic senescence.

From a diagnostic perspective, the meninges represent a promising and still underexplored source of biomarkers for cerebral senescence. Alterations in meningeal immune cell composition, cytokine profiles, lymphatic function, and fibrotic remodeling may serve as measurable indicators of age-related CNS dysfunction. Advances in imaging techniques, molecular profiling, and minimally invasive sampling approaches may facilitate the detection and monitoring of these changes in clinical and research settings.

Future research efforts should prioritize integrated anatomical and molecular investigations of the meninges across the lifespan. In particular, combining high-resolution structural analyses with immunohistochemical, transcriptomic, and proteomic approaches may help delineate the temporal sequence and mechanistic drivers of meningeal aging. Such studies are essential for distinguishing primary age-related alterations from secondary changes associated with neurodegenerative pathology.

Moreover, a deeper understanding of the interactions between meningeal immune cells, vascular and lymphatic networks, and ECM components may reveal novel targets for diagnostic stratification and therapeutic intervention. Longitudinal and comparative studies will be especially valuable for identifying early meningeal alterations that precede overt neurological decline, thereby enabling earlier detection and monitoring of cerebral aging.

## 5. Conclusions

As a summary, the evidence synthesized in this narrative review underscores the meninges as a dynamic and biologically active compartment that undergoes significant remodeling during aging. Anatomical changes, immunosenescence, lymphatic dysfunction, and ECM alterations collectively contribute to impaired CNS homeostasis and increased vulnerability to neurodegenerative processes. By integrating current knowledge and highlighting critical gaps, this review provides a conceptual framework for future anatomical and molecular studies of meningeal aging. Ultimately, advancing our understanding of meningeal biology may support the development of novel diagnostic biomarkers and improve the assessment of cerebral senescence and age-related neurological disorders.

Highlights and Unresolved Questions

- The precise temporal sequence of immune, lymphatic, and stromal remodeling events in the aging meninges remains incompletely defined.
- The causal relationships between meningeal immunosenescence, impaired glymphatic–meningeal lymphatic clearance, and parenchymal neurodegeneration require further mechanistic validation.
- The extent to which meningeal aging signatures are disease-specific versus reflective of generalized systemic aging remains unclear.
- Standardized, non-invasive biomarkers for assessing meningeal immune and clearance dysfunction in humans are currently lacking.
- The translational potential of imaging-based and molecular meningeal biomarkers for early diagnosis and longitudinal monitoring of cerebral senescence has yet to be established.
- Interventional strategies targeting meningeal immune remodeling, fibrosis, or lymphatic dysfunction remain largely unexplored.
- The translational validity of meningeal aging mechanisms identified in animal models, and their applicability to human cerebral senescence, remains incompletely established.

## Figures and Tables

**Table 1:** The meningeal immune cell populations and their role in inflammation and, respectively, in aging.

Meningeal Layer	Immune Cell Population	Physiological Role (Steady State)	Age-Related Changes (Immunosenescence)	Potential Diagnostic Relevance	References
Dura mater	Border-associated macrophages (BAMs)/CNS-associated macrophages	Immune surveillance; phagocytosis of pathogens and cellular debris; maintenance of tissue homeostasis	Shift toward monocyte-derived inflammatory macrophages; increased cytokine production; altered antigen presentation	Indicators of meningeal inflammaging and altered immune surveillance	[64,65,66]
	T lymphocytes (CD4^+^, CD8^+^, γδ, double-negative)	Regulation of immune surveillance; support of cognitive and behavioral functions	Increased accumulation and retention; elevated IFN-γ production	Cytokine profiling as a marker of impaired meningeal lymphatic drainage	[30,55,67,68]
	B cells and plasma cells	Local antibody production; immune modulation	Expansion of antibody-secreting populations; altered humoral responses	Potential markers of chronic meningeal immune activation	[29,69,70]
	DCs	Antigen presentation; initiation of adaptive immune responses	Enhanced activation and inflammatory signaling	Reflect altered antigen presentation dynamics in aging	[31]
	ILCs	Cytokine secretion; maintenance of immune balance	Functional dysregulation; pro-inflammatory bias	Indicators of innate immune imbalance	[29,71,72]
Arachnoid mater	—	Barrier function via tight junctions; restricted immune cell trafficking	Barrier permeability largely preserved; indirect effects of fibrosis and inflammation	Structural integrity relevant for CSF dynamics	[73,74]
Subarachnoid space	BAMs; T and B lymphocytes; DCs	Immune surveillance; phagocytosis; antigenpresentation	Reduced clearance due to impaired lymphatic drainage; accumulation of inflammatory mediators	Markers of impaired CSF immune clearance	[75,76,77]
Pia mater	Pial macrophages (BAMs)	Neurovascular regulation; immune surveillance	Enhanced local inflammatory responses; altered signaling with microglia	Indicators of neuroimmune interface dysfunction	[73,78]

BAMs: border-associated macrophages; CNS: central nervous system; DCs: dendritic cells; IFN-γ: interferon-beta; ILCs: innate lymphoid cells.

## Data Availability

No new data were created or analyzed in this study. Data sharing is not applicable to this article.

## Abbreviations

The following abbreviations are used in this manuscript:

BAMs	Border-Associated Macrophages
BBB	Blood–Brain Barrier
CNS	Central Nervous System
CSF	Cerebrospinal Fluid
DCs	Dendritic Cells
ECM	Extracellular Matrix
IFN-γ	Interferon-gamma
ILCs	Innate Lymphoid Cells
ISF	Interstitial Fluid
MMA	Middle Meningeal Artery
ROS	Reactive Oxygen Species
SAS	Subarachnoid Space
TGF-β	Transforming Growth Factor-beta
